# Primary Cervical Carcinosarcoma: Report of a Rare Case

**DOI:** 10.1055/s-0042-1744152

**Published:** 2022-08-02

**Authors:** Georgios Tsatsaris, Zacharias Fasoulakis, Antonios Koutras, Thomas Ntounis, Athina A. Samara, Athanasios Syllaios, Alexandros Diamantis, Maria Kouroupi, Charilaos Stamos, Emmanuel N. Kontomanolis

**Affiliations:** 1 Department of Obstetrics and Gynecology, Democritus University of Thrace, Dragana, Alexandroupolis, Greece; 2 Department of Obstetrics and Gynecology, National and Kapodistrian University of Athens, General Hospital of Athens “ALEXANDRA,” Athens, Greece; 3 Department of Surgery, University Hospital of Larissa, Larissa, Greece; 4 Department of Surgery, National and Kapodistrian University of Athens, Laikon General Hospital, Athens, Greece; 5 Department of Pathology, Democritus University of Thrace, Dragana, Alexandroupolis, Greece

**Keywords:** carcinosarcoma, cervical cancer, malignant mixed Müllerian tumors

## Abstract

**Background** Carcinosarcomas are malignant mixed Müllerian tumors (MMMT), containing both epithelial and mesenchymal components. Carcinosarcomas of the uterine cervix comprise an extremely rare histopathological entity, with less than 150 cases reported in the literature to date.

**Materials and Methods** A 79-year-old postmenopausal female patient was referred to our gynecological department due to a pelvic mass and vaginal bleeding. A cervical curettage was performed and the histological report revealed a malignant neoplasm with high cellularity consisting of two components; the first was a chondrosarcoma and the latter a adenocarcinoma. A diagnosis of MMMT was confirmed through immunohistochemical (IHC) staining. Neoadjuvant chemotherapy and radiotherapy were implemented, and a year later the patient underwent a radical hysterectomy and oncological pelvic lymph node dissection. She remains disease-free 12 months postoperatively.

**Conclusion** Primary cervical carcinosarcomas are extremely rare tumors demonstrating a bipartite profile. Preoperative diagnosis with appropriate immunochemistry testing of this rare entity is crucial to decision making.

Carcinosarcomas belong to a histological type of mixed malignant tumors originating from Müllerian (paramesonephric) ducts and the Wolffian (mesonephric) duct remnants, containing both epithelial and mesenchymal components. This specific type of cancer typically appears in the uterine corpus and represents 4 to 5% of uterine cancers worldwide. 1 Furthermore, about half of uterine sarcomas are carcinosarcomas with a poor prognosis and a 5-year survival rate of approximately 30%; for stage I patients the 5-year survival rate is nearly 50%. 2

Carcinosarcomas of the uterine cervix are a very rare histopathological entity with less than 150 cases reported in the literature to date. 1 Due to the relatively low frequency of the disease, most data available on the natural history of cervical carcinosarcomas are derived from case reports and small case series. In this context, therapeutic strategy as well as the prognosis, remains controversial.

Herein, we report a case of a 79-year-old patient diagnosed with a cervical carcinosarcoma, who underwent a radical hysterectomy after receiving neoadjuvant therapy.

## Case Report

A 79-year-old postmenopausal female patient was referred to our gynecological department due to vaginal bleeding. From her gynecological history, she was gravida 3, para 3, and her last menstrual period was at the age of 52. There was no family history of malignancies, while her past medical history included arterial hypertension, an appendectomy at the age of 10, and a left oophorectomy for a teratoma at the age of 22. Following her three labors, she never had an annual gynecological examination or screening.

Upon vaginal examination an erythematous and hemorrhagic mass was protruding out of the vagina, while digital examination of the rectus revealed no involvement of the parametria, vaginal, or pelvic wall. Laboratory values were in normal ranges and tumor markers (including Ca-125) were not increased. A magnetic resonance imaging of the pelvis was performed and a cervical mass with a diameter of 4 cm was revealed, without pelvic metastasis or lymph node infiltration. A positron emission tomography (PET scan) demonstrated a slightly enhanced uptake of gadolinium, which appeared in the cervical mass. There was no infiltration of the uterus or vaginal wall, and no other evidence of metastasis was observed. Based on these findings, the patient was diagnosed with a stage IIA2 according to the International Federation of Obstetrics and Gynecology (FIGO).

**Fig. 1:**
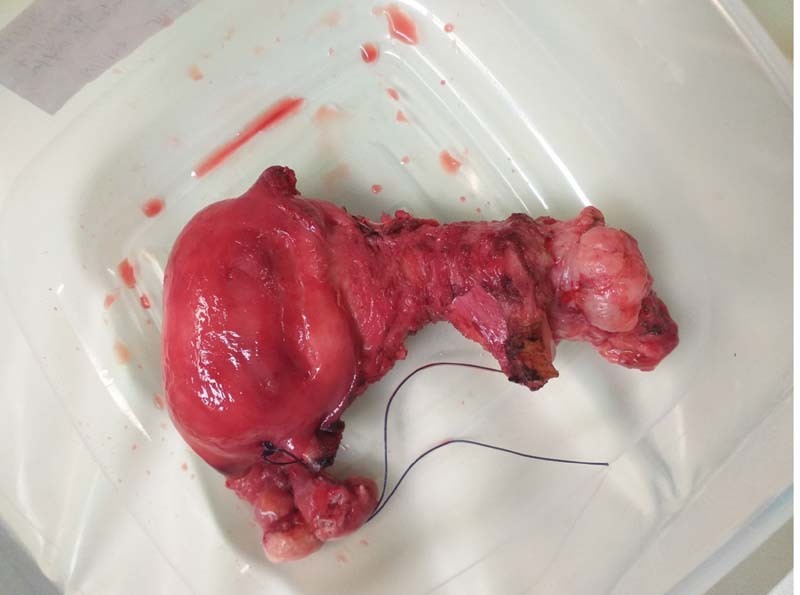
Surgical specimen of the uterus.

A cervical curettage was performed revealing a malignant neoplasm with high cellularity consisting of two components: the first was a chondrosarcoma and the latter a adenocarcinoma. The final diagnosis of a malignant mixed Müllerian tumor (MMMT) was confirmed with immunohistochemistry testing ( Fig. 1 ) for the epithelial markers (LMWCKs and CK7) and mesenchymal markers (Vimentin and S-100).

Based on these findings, a multidisciplinary oncological team suggested the administration of neoadjuvant therapy for better locoregional control, to shrink the tumor, and to increase the chance for complete resection with clear surgical margins, which is a prerequisite for cure. Neoadjuvant chemotherapy with 75 mg/m ^2^ of cisplatin, 5 g/m ^2^ of ifosfamide, and 50 mg/m ^2^ of paclitaxel, followed by eternal beam radiotherapy with 50 Gy in 25 fractions were administered. The course of neoadjuvant chemotherapy was repeated every 3 weeks with one course every week.

**Fig. 2:**
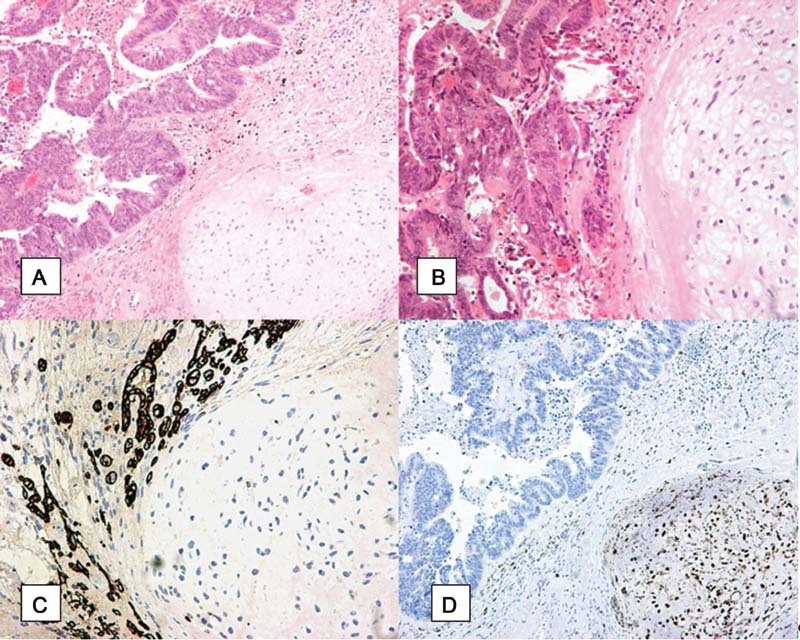
( **A** ) Poorly differentiated epithelial adenocarcinoma and sarcomatous heterogenous component/chondrosarcoma grade I (Hematoxylin-eosin, magnification*10), ( **B** ) poorly differentiated epithelial adenocarcinoma and sarcomatous heterogenous component/chondrosarcoma grade I (Hematoxylin-eosin, magnification*20), ( **C** ) immunohistochemical positivity of the epithelial component for CK7(magnification*20), ( **D** ) immunohistochemical positivity of the mesenchymal component for S-100 (magnification *10).

After completion of the neoadjuvant scheme, the patient was diagnosed as positive for SARS-CoV-2 (COVID-19) leading to a delay in the oncological operation. One year later, the patient underwent a radical hysterectomy with a right salpingo-oophorectomy and oncological pelvic lymph node dissection ( Fig. 2 ). The patient had an uneventful postoperative hospital stay and was discharged on the 5th postoperative day.

Histopathology examination revealed a high-grade bipartite tumor consisting of two components: a carcinomatous and a sarcomatous one. There was a medium differentiation adenocarcinoma consisting of medium- to large-sized neoplastic cells, arranged in an glandular pattern. Several sections unveiled a neoplasm with moderate differentiation ( Fig. 2 ), whereas in other sections the neoplasm was poorly differentiated. Regarding the mesenchymal component, there was a heterologous/cartilaginous element with focal atypia and sporadic presence of intracytoplasmic hyaline globules.

The immunohistochemical (IHC) examination revealed positivity for the markers S-100, vimentin, and p53 in the mesenchymal component. There was also MNF116, EMA, p53 positivity, and focal moderate positivity for the markers CK7 and CAM 5.2 for the epithelial component. The expression of p53 was a mutant overexpressed pattern. The p53 marker was also performed and demonstrated strong expression on both components, for example adenocarcinoma and chondrosarcoma. Regarding the p16 stain, an immunohistochemically focal cytoplasmic positivity was observed; the p16 is not expressed in all MMMTs. In our patient p16 was negative based on the IHC results. The presence of only focal cytoplasmic positivity and the lack of diffuse positivity for p16 make an human papilloma virus (HPV)-related etiology unlikely; HPV-ISH is not available in our pathology department. It was not feasible to perform in situ hybridization or PCR. The ER, PR, and CEA stains were performed with a negative result. Based on these findings, the diagnosis of chondrosarcoma (S-100 strongly positive, please refer to Fig. 2A ) was verified; the final histopathological diagnosis was carcinosarcoma. The endometrium, right ovary, right salpinx, and all lymph nodes were disease-free.

Based on the negative surgical margins and negative lymph node status on the histopathology report, a multidisciplinary team determined there was no need for adjuvant chemotherapy, and the patient was introduced to a close follow-up schedule. One year postoperatively, the patient remains disease-free and has a close oncological follow-up.

## Discussion

Carcinosarcomas or mixed malignant Müllerian tumors are highly malignant tumors, comprising of two components: an epithelial poorly differentiated carcinoma and a mesenchymal homologous or heterologous component. 2 For more than 150 years, malignant neoplasms arising in the uterus composed of both epithelial and mesenchymal elements have been a subject of debate. A mixed mesodermal tumor was first recognized in 1852 and was called “enchondroma.” 3

MMMT affects female patients with a wide age range spanning between 12 and 93 years. It is typically observed in postmenopausal women with a median age of approximately 65 years. About 76% of patients are of Caucasian origin and typically present with postmenopausal bleeding. 4 The most important risk factors include obesity, radiation therapy of the pelvis, increased age and infection with HPV, especially with types 16 and 18. More specifically, Grayson et al reported the presence of HPV DNA in all patients from a case series of eight carcinosarcoma cases. 5 Nearly 66% of patients were diagnosed with stage I or II, but the majority of them were diagnosed at stage IB of the disease. 5

Characteristics of the mesenchymal sarcomatous element of uterine carcinosarcomas (UCS), including the presence and type of heterologous components, have no relation with the existence of metastatic sites and vascular invasion. 6 De Jong et al 6 proved the monoclonal origin of the disease by investigating the tumor markers that are highly expressed, such as p53, MSH2, and MSH6 between carcinomatous and sarcomatous components. Upon analyzing the clinical features of carcinosarcomas, they concluded that non-endometrioid epithelial elements are related to metastases, myometrial invasion, lymphovascular infiltration, and isthmic and cervical involvement. 6 To improve prognosis, if the biological behavior of UCS is determined by the epithelial part of a bifacial tumor, treatment should focus on the epithelial tumor component of UCS in these malignancies. 6

UCS are typically developed by a combination of cellular masses that are diverged from a normal stem cell, or due to adjacent sarcomas and carcinoma collision. 2 There are four main theories regarding UCS histogenesis: the collision theory supporting the independent occurrence of mesenchymal and epithelial elements and their collision, giving the impression of a single mixed tumor; the combination theory claiming that one stem cell underwent divergence and differentiated during the early tumors' evolution, deriving both a sarcoma and a carcinoma; the conversion theory highlighting that during tumor evolution, carcinoma has as a result the sarcomatous element; and the composition theory suggesting a carcinoma in the endometrium with atypical and reactive connective tissue. 7

According to the conversion theory, most tumors appear to be originally monoclonal and then diverge and change abnormally from carcinomas to sarcomatous components. Both components (sarcoma and carcinoma) have various similar IHC markers, usually indicating the same X chromosome inactivation patterns, and share similar somatic mutations. 8 Other studies demonstrated that there are intensely shared patterns of heterozygosity loss, based on the use of polymorphic microsatellite markers in both components that indicate late patterns of deviating evolution. 9

Moreover, the most important factor associated with carcinosarcoma is former exposure to radiation. Varela-Duran et al suggest that post-irradiation carcinosarcomas occur at a younger age in relation to those newly arising. 10 Other important factors associated with carcinosarcoma development include exogenous estrogen, tamoxifen, and obesity.

Due to the rarity of the disease, there is no consensus regarding treatment. In general, patients are treated with a radical hysterectomy and with pelvic lymphadenectomy, in combination with radiotherapy and chemotherapy. In addition, despite numerous drug trials, only three chemotherapeutic agents were proven effective against carcinosarcoma: cisplatin, ifosfamide, and paclitaxel. 10 11 Finally, Homesley et al proved that overall response and survival rates were enormously improved due to the addition of paclitaxel in the treatment process. 12 A Cochrane review concludes that a combination of chemotherapy agents improves survival. 13 More specifically, the combination of ifosfamide and paclitaxel was associated with a significant improvement in overall and progression-free survival. The combination of cisplatin and ifosfamide offers a small improvement in progression-free survival over ifosfamide alone; however, the added toxicity may not justify the use of this combination. 14

With limited data from randomized trials available, the role of postoperative radiotherapy delivered as external-beam radiotherapy (EBRT), vaginal brachytherapy (VBT), or a combination of both in the management of carcinosarcoma of the uterus is not clearly defined. Findings from a recently published review report that survival effects of radiotherapy apparently persist when given in addition to chemotherapy. Whereas some studies see the strongest survival effects in patients with positive lymph nodes; propensity-score matched data indicate an overall survival effect of radiotherapy (EBRT + VBT or VBT alone) in FIGO stages I to III, regardless of lymph node surgery. 14

Neoadjuvant or preoperative chemotherapy is an alternative treatment option for patients who are not suitable for primary surgical treatment, either due to locoregional advanced disease or due to the patient's status. 15 The role of neoadjuvant chemotherapy has been studied in various types of gynecological malignancy, including ovarian and high-grade endometrial cancers. 15 16 17 However, the effectiveness of neoadjuvant chemotherapy in UCS has not been studied, likely due to the rarity of disease. In the only available retrospective cohort study of patients receiving neoadjuvant therapy for a stage IV UCS, with carboplatin/paclitaxel being the most commonly used regiments, there was no statistically significant difference in survival compared with patients who underwent surgery first. 17 To the best of our knowledge, our case represents the first case with a stage II UCS receiving neoadjuvant therapy with promising results.

Staging is crucial in decision making regarding treatment strategy, as it seems that the disease stage was an independent factor for recurrence and mortality. For the first two stages (I and II), a radical abdominal hysterectomy is preferred; in case of abdominal dissemination, omentectomy and bilateral salpingo-oophorectomy is the procedure of choice and adjuvant therapy may be required. Conversely, when a patient is at an advanced disease stage (III and IV), neoadjuvant therapy is preferred along with radical surgery and in some cases adjuvant therapy. 7 Radiotherapy is used to reduce local recurrence of a carcinosarcoma, in cases where surgical sites are inaccessible, remaining postoperative disease, and/or in positive lymph node status. 11

Due to the lack of large case series, prognosis of these patients is not well-known yet. Wolfson et al suggest that the most pivotal of all prognostic factors is the disease's surgical stage at the time of diagnosis. 18 Moreover, Amant et al report that carcinosarcomas will metastasize to the lymph nodes and the lungs in comparison to endometrial neoplasm. 19

## Conclusion

Primary cervical carcinosarcomas are extremely rare tumors with a biphasic profile. They consist of a carcinomatous and a mesenchymal component. Even though they exhibit several similarities with those that appear in the uterine corpus, they may have a better prognosis. Less than 100 cases have been reported worldwide and the limited bibliography makes it challenging to identify information regarding the best way to treat these neoplasms. Approximately 50% of neoplasms eventually reappear, scientific research progresses to achieve more effective modalities of cancer remedies. After having completed aggressive surgery, neoadjuvant chemotherapy and radiotherapy, the patient remains disease-free.
